# FLEXc: protein flexibility prediction using context-based statistics, predicted structural features, and sequence information

**DOI:** 10.1186/s12859-016-1117-3

**Published:** 2016-08-31

**Authors:** Ashraf Yaseen, Mais Nijim, Brandon Williams, Lei Qian, Min Li, Jianxin Wang, Yaohang Li

**Affiliations:** 1 Department of Electrical Engineering & Computer Science, Texas A&M University-Kingsville, Kingsville, TX 78363 USA; 2 Department of Mathematics & Computer Science, Fisk University, Nashville, TN 37208 USA; 3 School of Information Science and Engineering, Central South University, Changsha, 410083 China; 4 Department of Computer Science, Old Dominion University, Norfolk, VA 23529 USA

## Abstract

**Background:**

The fluctuation of atoms around their average positions in protein structures provides important information regarding protein dynamics. This flexibility of protein structures is associated with various biological processes. Predicting flexibility of residues from protein sequences is significant for analyzing the dynamic properties of proteins which will be helpful in predicting their functions.

**Results:**

In this paper, an approach of improving the accuracy of protein flexibility prediction is introduced. A neural network method for predicting flexibility in 3 states is implemented. The method incorporates sequence and evolutionary information, context-based scores, predicted secondary structures and solvent accessibility, and amino acid properties. Context-based statistical scores are derived, using the mean-field potentials approach, for describing the different preferences of protein residues in flexibility states taking into consideration their amino acid context.

The 7-fold cross validated accuracy reached 61 % when context-based scores and predicted structural states are incorporated in the training process of the flexibility predictor.

**Conclusions:**

Incorporating context-based statistical scores with predicted structural states are important features to improve the performance of predicting protein flexibility, as shown by our computational results. Our prediction method is implemented as web service called “FLEXc” and available online at: http://hpcr.cs.odu.edu/flexc.

## Background

At the molecular level, protein dynamics and flexibility are vital elements for understanding protein functions. The structural flexibility of proteins enables their motion, which is associated with numerous biological activities such as molecular recognition [1–3], allosteric regulation [4–6], catalytic activity [7, 8], and protein stability [9, 10].

Conformational changes driven by protein flexibility and dynamics are considered the basis of misfolding, which is responsible for intrinsic disorders. In fact, the recent discovery of the significance of disordered proteins in the last few years has intensely increased the interest in protein flexibility [11–16].

Consequently, information on protein flexibility is as important as tertiary structure to provide more insights into understanding protein function, and consequently will have significant impact on genomic study, disease research, and drug-design [17].

B-factors (also referred to as B-values, Debye-Waller factors, or temperature factors) reported in experimentally determined protein structures are commonly used to represent protein flexibility and its local mobility [18, 19]. They indicate both the static mobility, related to the molecule orientation, and dynamic mobility, caused by the atoms vibration [20, 21].

The B-factor is given by,

$$ {\mathrm{Bfactor}}_{\mathrm{i}} = 8{\uppi}^2{{\mathrm{U}}_{\mathrm{i}}}^2 $$

where ${{\mathrm{U}}_{\mathrm{i}}}^2$ is the mean-squares displacement of atom *i*. The values of the B-factors are usually between 15 to 30 Å^2^, and sometimes higher than 30 for more flexible regions.

Although each atom in protein atomic resolution structures has its B-factor, the B-factor of the whole residue is generally represented by its Cα B-factor. The residues with low B-factor values are usually more stable in structure than the ones with large B-factor values.

A variety of approaches have been proposed by different groups to predict protein flexibility, mainly dealing with the so called “classification problem”. Protein residues are classified into two states as rigid or flexible on the basis of a B-value threshold [21, 22]. Others extended the classification into three states (rigid, intermediate, and flexible) [23]. Some other approaches also provide real value prediction [24]. Methods have been developed using different protein datasets and different computational algorithms, including logistic regression [25], support vector regression [22, 24, 26], and neural networks [21]. Generally, flexibility prediction methods define flexibility through Cα B-factor obtained from experimental data, such as PROFbval [21] and PredBF [26]. Other methods use different descriptors of flexibility, such as CamP [27] which uses protection values gained by equilibrium hydrogen exchange experimentations. PredyFlexy [23] examines flexibility based on two descriptors, the root mean square fluctuations obtained by running molecular dynamics simulations and the B-factor values.

When dealing with the classification problem, features influencing the flexibility of residues such as evolutionary information revealed by multiple sequence alignments are encoded as input in the machine learning methods in order to enhance the prediction performance. Hence, extracting then selecting good features is key to the accuracy and overall performance of the machine learning algorithms. Most flexibility prediction methods are based on protein sequence and evolutionary information, predicted secondary structures and/or solvent accessibility for their encodings [21–27].

The flexibility state of a residue is frequently correlated with the flexibility states of its neighbors. In other words, the flexibility states of the neighbors are very effective features for predicting the flexibility state of a residue. For example, if both neighboring residues are rigid, then the residue in the middle is more likely to be rigid, and vice versa. Unfortunately, we can’t use the true flexibility states as features since they are not known in advance. However, the likelihood of a residue adopting a specific flexibility state may also be an important feature.

Moreover, previous studies have shown that there is a strong correlation among flexibility, secondary structures, and solvent accessibility [21]. Hence, encoding information about residues structural features will also enhance the prediction accuracy.

In this work, we examine flexibility according to the experimentally determined B-factors. We then define 3 flexibility states and propose a neural network based method for predicting protein flexibility along the amino acid sequence. We describe the approaches of extracting statistical scores to measure the favorability of residues’ flexibility in presence of its surrounding neighbors in sequence from a large training dataset based on the mean-field potentials [28]. These approaches were successfully applied in our previous work for predicting protein disulfide bonding [29], secondary structures [30, 31], and solvent accessibility. The basic idea is based on the observation that residues’ flexibility exhibit strong local dependency. We derive statistics for residues as singles, doubles, and triples in a sequence window from protein structures found in PDB [32]. Then scores measuring the pseudo-energy of a residue adopting specific flexibility state are determined using the potentials of mean force approach. These scores are then integrated with data from multiple sequence alignments, predicted secondary structure and solvent accessibility states, and amino acid properties to train neural networks for flexibility prediction. An analysis of the relationship between flexibility and residues’ structural features (secondary structures and solvent accessibility) is further discussed.

7-fold cross validations are performed. Benchmark datasets are used to further validate and demonstrate the effectiveness of our approach. Finally, the benchmarks are also used to compare our method with a set of popularly used methods for flexibility prediction. A web server named “FLEXc” hosting our method is currently available online at http://hpcr.cs.odu.edu/flexc.

## Methods

### Protein data sets

Two protein datasets including Cull16633 and Cull5547 generated by the PISCES server [33] are used in this work. Cull16633 is used to generate context-based statistics. It contains 16633 proteins with 50 % (at most) sequence identity and a resolution cutoff of 3.0 Å. Cull5547 is used for neural network training and testing. It includes 5547 proteins with 25 % (at most) pair-wise sequence identity and a resolution cutoff of 2.0 Å.

PSI-BLAST [34] is used to produce Position Specific Scoring Matrix (PSSM) data for all protein chains in our dataset. PSSM data is used in producing statistics from Cull16633 and in input encoding of Cull5547 for neural network training. Short chains (<40 residues) are removed because PSI-BLAST is usually incapable of generating profiles for very short sequences. We also eliminate residues with undetermined flexibility state from the dataset. The total number of protein chains after filtering is 5271.

The absolute B-factor values are determined from the proteins’ PDB files. The secondary structure assignments and solvent accessibility values of the residues in the training dataset are produced by the DSSP program [35].

The recent CASP11 targets as well as the previous CASP10, CASP9, and CASP8 targets [36] are used for benchmarking our prediction method. Hence, any sequence with more than 25 % similarity in sequence with any other sequence in the benchmarks is removed from the Cull16633 and Cull557 when context-based scores are generated and when neural networks are trained.

### Normalized B-values

In a protein PDB format file, every atom has a B-factor value in the ATOM records. B-factors of Cα atoms are used to represent the B-factors of the residues. We extracted Cα B-factors of the protein chains from their PDB files. The raw values are then normalized, since B-factors from different protein structures are on different scales [22]. The following normalization is applied:

$$ {\mathrm{Bfactor}}_{\mathrm{normalized}}=\raisebox{1ex}{$\left({\mathrm{Bfactor}}_{\mathrm{raw}}-\upmu \right)$}\!\left/ \!\raisebox{-1ex}{$\upsigma $}\right. $$

where μ is the mean of B-factors of a given structure, and σ is the standard deviation.

**Fig. 1:**
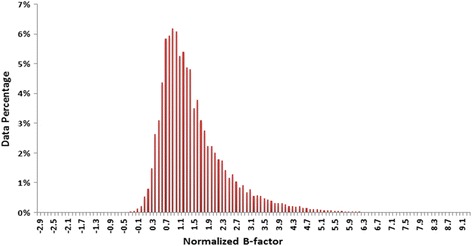
Distribution of the normalized B-factors in Cull5547. Large normalized B-values (to the right) indicate more flexible residues and small normalized B-values (to the left) indicate more rigid residues. Most residues fall in the middle (intermediate flexibility)

The frequency distributions of the residues’ B-factors vary depending on their positions in the protein tertiary structure. The normalized B-factors of the protein residues in our dataset range from -2.9 to 12.8. Figure 1 shows the distribution of the normalized B-factors in the Cull5547 dataset.

### Features’ representation

A combination of sequence and structural information are used to represent protein residues. Each residue in our dataset is described by a vector of the following parameters:

#### Sequence and evolutionary information

Different amino acid types have different preferences for B-factor values. Similar to many studies that try to predict structural features from the protein sequence [29–31], we started with the sequence and we incorporate its evolutionary information in our method. The evolutionary information is represented by the PSSM data which is revealed by multiple sequence alignment (MSA) of a family of homologues proteins. This information forms the main input encodings to our neural network, trained to recognize and discriminate the different flexibility states. We use the PSI-BLAST program [34] with 3 iterations of searching against non-redundant sequence database (NR) to generate PSSM data for Cull5547 dataset.

#### Context-based scores

Apparently, the surrounding residues have strong influence on the chemical property of a residue in its flexibility state. In this work, similar to our previous work employed in DINOSOLVE [29], SCORPION [30], and CASA, we collect statistics of singlets (${\mathrm{R}}_{\mathrm{i}}$), doublets (${\mathrm{R}}_{\mathrm{i}}{\mathrm{R}}_{\mathrm{i}+\mathrm{k}}$), and triplets (${\mathrm{R}}_{\mathrm{i}}{\mathrm{R}}_{\mathrm{i}+{\mathrm{k}}_1}{\mathrm{R}}_{\mathrm{i}+{\mathrm{k}}_2}$) residues at different positions in protein chains in a window of size 7 residues ($-3\le k,{k}_1,{k}_2\le 3,\;k,{k}_1,{k}_2\ne 0$). These statistics represent approximations of the possibilities of residues adopting certain flexibility states when none, one, or two neighboring residues are considered. Based on the potentials of mean force method [28], the statistics are used to generate context-dependent pseudo-potentials that are then integrated as additional features in encoding our input for training the neural networks.

We calculate the mean-force potentials ${\mathrm{U}}_{\mathrm{singlet}}\left({\mathrm{R}}_{\mathrm{i}},{\mathrm{C}}_{\mathrm{i}}\right)$, ${\mathrm{U}}_{\mathrm{doublet}}\left({\mathrm{C}}_{\mathrm{i}},\;{\mathrm{R}}_{\mathrm{i}}{\mathrm{R}}_{\mathrm{i}+\mathrm{k}}\right)$ and ${\mathrm{U}}_{\mathrm{triplet}}\left({\mathrm{C}}_{\mathrm{i}},\;{\mathrm{R}}_{\mathrm{i}}{\mathrm{R}}_{\mathrm{i}+{\mathrm{k}}_1}{\mathrm{R}}_{\mathrm{i}+{\mathrm{k}}_2}\right)$ for a residue ${\mathrm{R}}_{\mathrm{i}}$ adopting flexibility state ${\mathrm{C}}_{\mathrm{i}}$. Then, the pseudo-potential for ${\mathrm{R}}_{\mathrm{i}}$ under its amino acid environment is

$$ \mathrm{U}\left({\mathrm{C}}_{\mathrm{i}}, \dots {\mathrm{R}}_{\mathrm{i}-1}{\mathrm{R}}_{\mathrm{i}}{\mathrm{R}}_{\mathrm{i}+1}\dots \right)={\mathrm{U}}_{\mathrm{singlet}}\left({\mathrm{C}}_{\mathrm{i}},\ {\mathrm{R}}_{\mathrm{i}}\right)+{\displaystyle \sum_{\mathrm{k}}}{\mathrm{U}}_{\mathrm{doublet}}\left({\mathrm{C}}_{\mathrm{i}},\ {\mathrm{R}}_{\mathrm{i}}{\mathrm{R}}_{\mathrm{i}+\mathrm{k}}\right)+{\displaystyle \sum_{{\mathrm{k}}_1,{\mathrm{k}}_2}}{\mathrm{U}}_{\mathrm{triplet}}\left({\mathrm{C}}_{\mathrm{i}},\ {\mathrm{R}}_{\mathrm{i}}{\mathrm{R}}_{\mathrm{i}+{\mathrm{k}}_1}{\mathrm{R}}_{\mathrm{i}+{\mathrm{k}}_2}\right) $$

#### Protein structural features

Residues’ flexibility is strongly correlated with secondary structures and solvent accessibility. Regular secondary structure elements such as alpha helices and beta strands tend to be more stable than random coils. Buried segments tend to be less flexible than exposed ones. Consequently, incorporating structural features with sequence information will significantly enhance the performance of the predictor.

Predicted structural features are incorporated in our method. We use the methods SCORPION [30] and CASA for secondary structure and solvent accessibility predictions, respectively.

#### Amino acid properties

We also use five amino-acid properties for encoding [37]: a steric parameter (graph shape index), polarizability, volume, hydrophobicity, and isoelectric point.

### Threshold selection

Some prediction methods consider only two flexibility classes and some others consider three classes. Defining thresholds to discriminate between classes of flexibility is rather arbitrary and subjective in many studies [18, 19, 21–24]; mainly attributed to the differences in the training datasets, computational methods, and flexibility descriptors. Studies that use same flexibility descriptor and similar computational methods for predicting flexibility base their threshold selection on the dataset, such that the number of training samples in the different classes defined for flexibility is balanced [21–24].

In this work, we define three classes with thresholds (-1.1, 2.2); a normalized B-factor value of less than -1.1 is considered rigid, a value greater than 2.2 is considered flexible, otherwise the residue is considered to be in intermediate state. A two state classification is also defined in this work in order to compare our method with previous work. A threshold value of 0.03 is used in one experiment and a value of -0.3 is used in another one.

### Neural network model

Our method incorporates one phase of neural network training. The standard feed-forward back-propagation architecture was adopted with 250 hidden nodes. We selected a window of 15 residues long where the neural network is trained to predict the flexibility state of the residue in the center of that window. Different settings for our method were tested and the chosen settings correspond to the optimal obtained results.

**Fig. 2:**
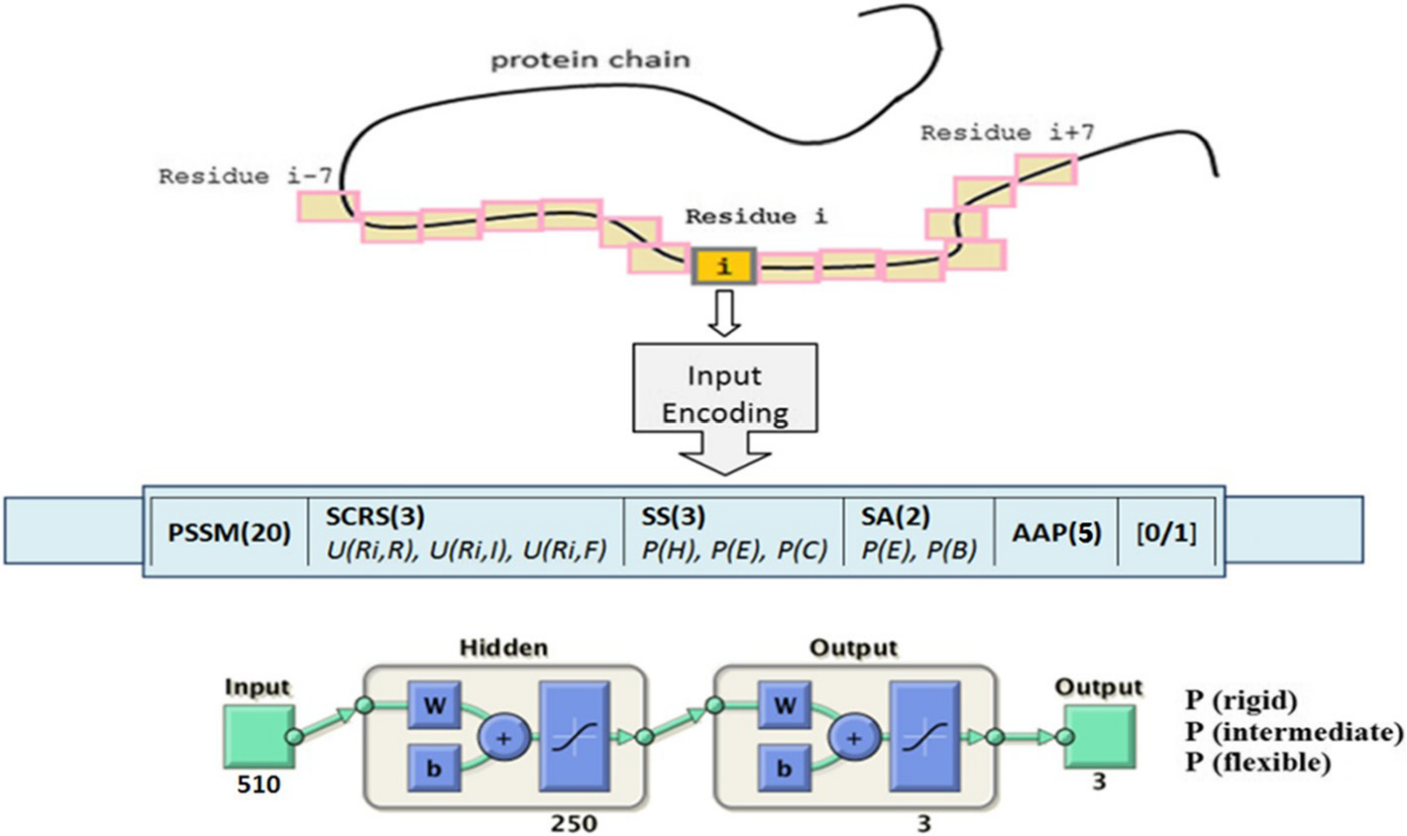
Encoding and neural network architecture for flexibility prediction. PSSM(20): position specific scoring matrix. SCRS(3): context-based scores of residue R_i_ in Rigid, Intermediate, and Flexible states. SS(3): predicted secondary structures, represent probabilities of residue R_i_ in Helix, Sheet, and Coil. SA(2): predicted relative solvent accessibility, represent probabilities of residue R_i_ in Extended and Buried states. AAP(5): amino acid properties

Twenty values for PSSM data, 3 values for context-based scores, 3 values for predicted secondary structures, 2 values for predicted solvent accessibilities, 5 values for amino acid properties, and 1 value to specify C-terminals or N-terminals overlap are used to represent each residue. A total of 510 input values are used to encode a residue in 3-state flexibility prediction. Figure 2 shows the neural network input encoding and the architecture of our flexibility prediction method.

### Cross validation

For reliable assessment of our method’s performance, the N-fold cross validation is used on Cul5547 dataset, where *N*=7. The protein sequences in the training set are divided into 7 subsets. At each stage, 5 subsets are selected for training whereas the other 2 subsets are selected for neural network testing and validation, separately. The process is repeated 7 times (folds) and the overall accuracy of the prediction is calculated as the average of the accuracies obtained from the 7 folds.

### Performance evaluation

For the evaluation, we calculate the prediction rate by dividing the number of residues that were predicted correctly (TP) over the total number of residues (N). I.e. Q = TP/N. In order to compare our method with some previous methods, we also compute the F-measure [25], as F = 2*A*C/(A+C), such that A stands for accuracy is defined by A=TP/(TP+FP) and C stands for coverage is calculated as C=TP/(TP+FN). TP (True Positive) represents the residues predicted correctly to be flexible, TN (True Negative) represents the residues predicted correctly to be not flexible, FN (False Negative) represents the residues predicted to be rigid but observed to be flexible, and FP (False Positive) represents the residues predicted to be flexible but observed to be rigid.

## Results

The evolutionary information of protein sequences combined with the context-based flexibility scores, predicted structural features that we found to be correlated with flexibility, and amino acid properties enhanced the accuracy of our method by 8.4 % over the prediction with evolutionary information only. The overall Q_3_ accuracy of our method reached 61 %.

Table 1 compares the 7-fold cross validated qualities of protein flexibility predictions based on different encoding schemes: PSSM-only encoding, PSSM+context-based statistical scores encoding, and all-features encoding. Q_R_, Q_I_, and Q_F_ measure the quality of predicting the rigid state, intermediate state, and flexible state, respectively. Q_3_ measures all 3-state prediction accuracy. Compared to the prediction method trained with PSSM-only encoding, the method incorporating statistical scores as additional features along the PSSM data for encoding results in enhancements of 4.7 % in the Q_3_ accuracy. On the other hand, the neural network trained with all features described in section 2 results in more significant improvements. Table 2 also shows the accuracy improvement of incorporating context-based scores with PSSM encoding and the improvement of all-features’ encoding over PSSM only on the CASP8-11 targets.

**Table 1:** Prediction performance on Cull5547 dataset

	Q_R_	Q_I_	Q_F_	Q_3_
PSSM Only	56.7	50.4	51.3	52.6
PSSM+Scores	57.5	56.0	58.9	57.3
All-features (FLEXc)	61.7	57.2	66.6	61.0

Comparison of prediction accuracy using PSSM-only encoding, PSSM+context-based scores encoding, and all-features encoding on Cull5547 using 7-fold cross validation. All-features including PSSM, context-based scores, predicted secondary structures and solvent accessibility, and amino acid physicochemical properties

**Table 2:** Prediction performance on benchmark datasets

	CASP11	CASP10	CASP9	CASP8
PSSM Only	47.1	48.6	50.8	50.7
PSSM+Scores	52.6	52.7	52.9	52.6
All-features (FLEXc)	54.4	54.2	54.9	53.8

Comparison of Q_3_ prediction performance of protein flexibility using PSSM-only encoding, PSSM+context-based scores encoding, and all-features encoding on CASP8, CASP9, CASP10, and CASP11 targets

Different groups have used different computational methods, datasets, and flexibility descriptors to predict flexibility from protein sequence. Moreover, the selection of thresholds to define flexibility classes is neither objective nor optimal. As such, direct comparison between these methods is hard. However, we try to assess our prediction method by comparing the results to those presented by the popularly used methods. Public benchmarks including CASP11, CASP10, CASP9, and CASP8 targets are used to validate our method.

PredyFlexy [23] is a popular method for predicting flexibility with Q_3_ accuracy of 49.6 %, such that Q_R_, Q_I_, and Q_F_ are 47.4 %, 48.3 %, and 55 %, respectively. Our method exhibits higher performance measures over PredyFlexy.

Table 3 shows a comparison between FLEXc and PredyFlexy on the benchmark datasets. To ensure fairness in comparison, all homologues (with higher than 25 % sequence identity) to the sequences presented in those benchmarks are removed from our datasets when generating the statistical scores and when training the neural network. A significant improvement of ~12 %, in average, is achieved over PredyFlexy prediction method.

**Table 3:** Comparison of prediction performance of FLEXc with PredyFlexy on benchmarks of CASP(8-11) targets

Benchmark	Method	Q_R_	Q_I_	Q_F_	Q_3_
CASP11	PredyFlexy	41.5	41.3	58.3	42.0
	FLEXc	48.3	55.4	65.2	54.4
CASP10	PredyFlexy	36.4	42.5	53.5	42.4
	FLEXc	47.6	56.4	62.2	54.2
CASP9	PredyFlexy	37.9	42.0	57.4	42.3
	FLEXc	50.1	55.2	62.2	54.9
CASP8	PredyFlexy	40.3	41.4	55.6	41.8
	FLEXc	49.1	58.4	57.0	53.8

The PROFbval [21] method provides two states prediction. The states are defined according to a strict threshold of 0.03 and a non-strict threshold of -0.3. To compare our results with PROFbval, we modify our method to predict two states based on PROFbval thresholds. Bornot et al. [23] also modify PredyFlexy to provide 2-state prediction using B-factor as flexibility descriptor, and compare the results with PROFbval and PredBF [26].

Table 4 shows the comparison of our 2-state prediction results with 2-state PredyFlexy and PROFbval using F-measure. The results in Table 4 regarding the other methods are reported from [21, 23]. F-measures of 58.46 % and 72.8 % were obtained from our method for strict and non-strict thresholds, respectively, whereas PredyFlexy obtained 53.3 % and 71.9 %, and PROFbval method obtained 53.3 % and 71.9 %.

**Table 4:** Comparison of performance of 2-state FLEXc prediction with 2-state PredyFlexy and PROFbval prediction results using F-measure

	PredyFlexy	PROFbval	FLEXc
Strict, (threshold=0.03)	48.08	53.30	58.46
Non-Strict (threshold=−0.3)	71.99	71.90	72.80

Furthermore, Bornot et al. [23] reported F-measures of 52.9 % and 68.3 % using PredBF method with strict threshold of 2.3 and non-strict threshold of -1.4, respectively. Compared to the other methods, our results are very encouraging.

## Discussion

### Flexibility of secondary structure segments

We analyze the correlation of secondary structures with residues’ flexibility, and we found that residues in coil regions have higher B-values compared to the residues present in other regular secondary structure types (helix and strand). This observation is similar to previous studies in protein flexibility [21].

**Fig. 3:**
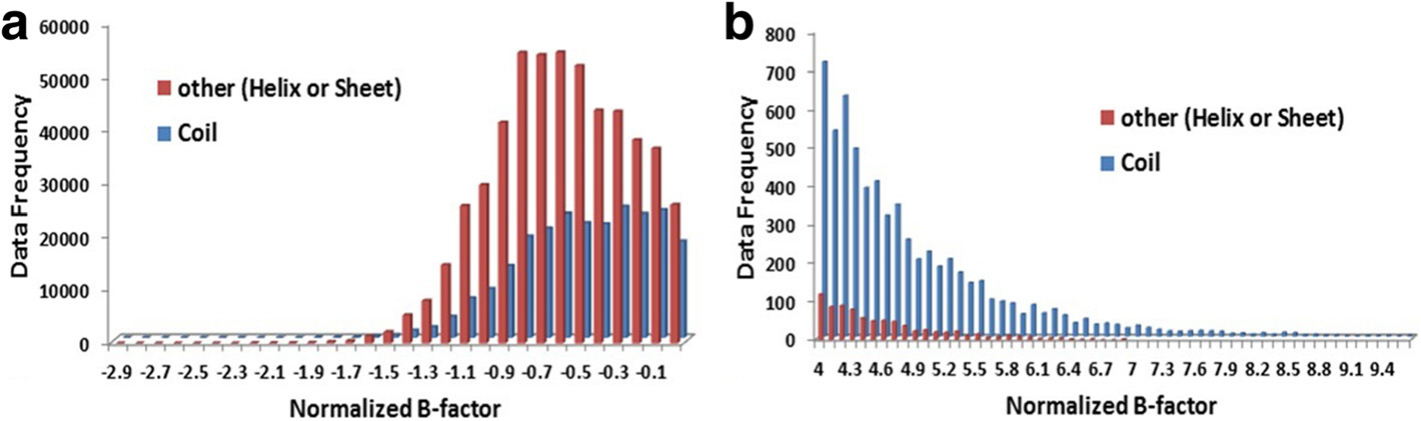
Frequency distribution of normalized B-factors associated with secondary structures assignment (coil and others) in Cull5547 dataset. **a** Distribution of B-factors from -2.9 to 0. **b** Distribution of B-factors from 4 to 9.4

Figure 3 shows the frequency distribution of B-values for Cull5547 residues in coils and other secondary structure types (helix and strand). The figure is plotted from the two ends of the normalized B-values to provide clearer view of the relationship between B-values and secondary structure. Figure 3a shows that larger number of residues with low B-factors is found in helixes and strands rather than in coils. On the other hand, Fig. 3b shows that residues with high B-values are more frequently found in coils.

The inclusion of the predicted secondary structure in our method improves the performance by ~4 % compared to the basic method of encoding PSSM data only.

### Flexibility of solvent accessible areas

**Fig. 4:**
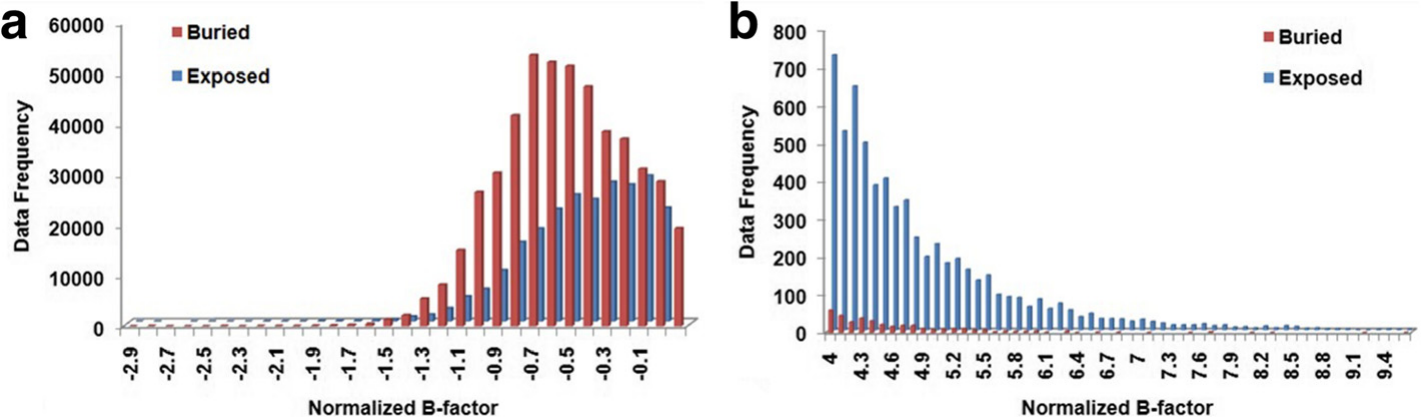
Frequency distribution of normalized B-factors associated with the relative solvent accessibility assignment (buried and exposed) in Cull5547 dataset. **a** Distribution of B-factors from -2.9 to 0. **b** Distribution of B-factors from 4 to 9.4

A similar analysis is done to understand the relationship between flexibility and residues’ exposure to solvent. We found that buried residues which are present in the protein core tend to have lower B-values and hence are more rigid than the residues present on the protein surface. Figure 4 shows the frequency distribution of the normalized B-values in correlation to the accessibility state. The figure is plotted from the two ends of the normalized B-values for clearer discussion. Figure 4a shows that residues with low B-values are more frequently found to be buried. Whereas, Fig. 4b shows that residues with high B-values are more likely to be exposed.

The inclusion of the predicted solvent accessibility states in our method improves the prediction performance by ~6 % compared to the basic method of encoding PSSM data only. In fact, solvent accessibility correlates rather well with flexibility than the case with secondary structure. However, both pieces of information are important to improve the prediction process.

### Flexibility and disordered regions

An important application of protein flexibility prediction is the study and prediction of intrinsically disordered proteins. In fact, the discovery of the significance of disordered proteins in the last few years has strongly driven the interest in studying protein flexibility. Intrinsically disordered proteins are typically associated with critical biological processes such as signaling and regulation [3, 38]. The correlation between protein disorder and flexibility is reported in some studies, and many disorder prediction methods currently incorporate flexibility in their implementations [25, 39]. Improving the flexibility prediction will greatly benefit the study of disorder protein regions.

## Conclusions

A new method for predicting flexibility in proteins is implemented. The method incorporates sequence and evolutionary information, context-based scores, predicted secondary structures and solvent accessibilities, and amino acid properties to predict protein flexibility. The context-based statistical scores are derived using the mean-field potentials method. An analysis of the correlation between protein flexibility, secondary structures, and solvent accessibility is discussed. The analysis presents the importance of incorporating structural features in the prediction method.

The effectiveness of our method, FLEXc, has been presented in the computational results of the 7-fold cross validations and the testing on benchmark datasets, where enhancements of prediction accuracies are observed. A comparison with popularly used methods is also provided such that our method shows higher prediction accuracies.

Even though the overall improvement of FLEXc over existing methods for predicting protein flexibility is relatively small, from protein tertiary prediction perspective, reducing even fractions of percent of inaccuracy will be very useful in protein modeling efficiency, mainly because the search space for finding a tertiary structure goes up superlinearly with the fraction of inaccuracy. Moreover, since our approach of calculating the sores depends on the number of known structures deposited in the PDB, with more structures being discovered, the PDB size will keep on increasing. This will enable us to obtain more accurate statistics and will provide a potential to achieve improvements in prediction accuracy in the future.
